# Bromodomain Inhibitors Modulate FcγR-Mediated Mononuclear Phagocyte Activation and Chemotaxis

**DOI:** 10.3389/fimmu.2022.885101

**Published:** 2022-05-10

**Authors:** Gemma D. Banham, Colin Y. C. Lee, John R. Ferdinand, Rebeccah J. Matthews, Chenzhi Jing, Nicholas Smithers, Rab K. Prinjha, Menna R. Clatworthy

**Affiliations:** ^1^ Molecular Immunity Unit, Department of Medicine, Medical Research Council Laboratory of Molecular Biology, University of Cambridge, Cambridge, United Kingdom; ^2^ Cellular Genetics, Wellcome Sanger Institute, Cambridge, United Kingdom; ^3^ Epinova DPU, Immuno-Inflammation Centre of Excellence for Drug Discovery, GlaxoSmithKline, Medicines Research Centre, Stevenage, United Kingdom

**Keywords:** BET inhibitors, systemic lupus erythematosus (SLE), Fcγ-receptor, dendritic cell chemotaxis, antibody-mediated inflammation

## Abstract

IgG antibodies form immune complexes (IC) that propagate inflammation and tissue damage in autoimmune diseases such as systemic lupus erythematosus. IgG IC engage Fcγ receptors (FcγR) on mononuclear phagocytes (MNP), leading to widespread changes in gene expression that mediate antibody effector function. Bromodomain and extra-terminal domain (BET) proteins are involved in governing gene transcription. We investigated the capacity of BET protein inhibitors (iBET) to alter IgG FcγR-mediated MNP activation. We found that iBET dampened IgG IC-induced pro-inflammatory gene expression and decreased activating FcγR expression on MNPs, reducing their ability to respond to IgG IC. Despite FcγR downregulation, iBET-treated macrophages demonstrated increased phagocytosis of protein antigen, IgG IC, and apoptotic cells. iBET also altered cell morphology, generating more amoeboid MNPs with reduced adhesion. iBET treatment impaired chemotaxis towards a CCL19 gradient in IC-stimulated dendritic cells (DC) *in vitro*, and inhibited IC-induced DC migration to draining lymph nodes *in vivo*, in a DC-intrinsic manner. Altogether, our data show that iBET modulates FcγR-mediated MNP activation and migration, revealing the therapeutic potential of BET protein inhibition in antibody-mediated diseases.

## Introduction

IgG antibodies propagate inflammation and cause tissue damage in a number of autoimmune diseases and in solid organ transplantation. Their pathogenicity is exemplified by systemic lupus erythematosus (SLE), a disease characterized by the deposition of autoantibody-containing immune complexes (IC) in tissues such as the skin or kidney (1). Many pro-inflammatory effects of IgG are mediated by binding to Fcγ receptors (FcγRs). These surface glycoproteins are expressed by many immune cells, including monocytes, macrophages and dendritic cells (DCs), collectively termed mononuclear phagocytes (MNPs). FcγRs may be activating (in humans, FcγRIIA, IIIA, IIIB), or inhibitory (FcγRIIB) (2, 3). Deficiency or dysfunction of FcγRIIB results in susceptibility to SLE in both mice and humans (3–8), highlighting the importance of this pathway in disease pathogenesis.

Almost all tissues contain a network of MNPs, poised to detect and respond to local immune challenges. Deposited IgG IC may be phagocytosed by MNPs in an FcγR-dependent manner and undergo degradation and processing for antigen presentation (2, 9–11), although non-myeloid cells such as B cells also have the capacity to process and present antigen (12). FcγR cross-linking on macrophages also results in the production of inflammatory cytokines that assist in pathogen clearance, but can potentially propagate tissue inflammation in autoimmune diseases (7, 13). In DCs, IgG IC promote maturation and the expression of co-stimulatory molecules required for immunogenic antigen presentation to CD4 T cells (14, 15). In addition to a requirement for maturation, tissue-resident DCs must be geographically re-located from peripheral tissues to lymph nodes to permit interactions with T cells (16–19). This re-location is enhanced by FcγR cross-linking (20) increasing the likelihood of encountering naïve CD4 T cells with the relevant antigen receptor within lymph nodes, potentially promoting autoimmune T cell activation if autoantigen-containing IC have been internalized. Indeed, abnormalities in DC function, including antigen presentation and chemokine receptor function, have been reported in SLE (21, 22). Therefore, both macrophages and DCs play differing but important roles in mediating autoantibody-associated inflammation and represent a useful therapeutic target in this context.

FcγR-dependent MNP activation results in profound changes in gene expression that mediate antibody effector function (23). In the past decade, there has been an increasing appreciation of the importance of epigenetic marks such as DNA methylation and histone modifications in determining whether genes are expressed. Bromodomain and extra-terminal domain (BET) proteins (BRD2, BRD3, BRD4 and BRDT) are chromatin ‘reader’ proteins that detect acetylated histones and govern the assembly of the chromatin complexes required for gene transcription (24, 25). Given the pivotal role of BET proteins in transcriptional regulation, small molecule synthetic histone mimics that inhibit binding of acetylated histones to BET proteins have been developed as potential anti-cancer and anti-inflammatory drugs (26–28). I-BET151 (GSK525762A), referred to hereafter as iBET, is a potent inhibitor of BRD2, BRD3 and BRD4 (29, 30), which are ubiquitously expressed proteins, including in myeloid cells (25, 31).

Treatment options in autoantibody-mediated disease such as SLE remain limited. B cell-targeted therapy may reduce antibody generation, but limiting FcγR-associated immune cell activation by existing autoantibody remains an unmet need. Inhibitors of BET proteins have shown some utility in animal models of autoimmunity (32) including those with proven antibody-dependent component, such as collagen-induced arthritis (33). These small molecule inhibitors has also been shown to selectively inhibit the transcription of a subset of inflammatory genes in macrophages following toll-like receptor (TLR)4 stimulation (34, 35), including MNP chemo-attractants in tissue injury such as CCL-2. Here, we explored the potential of BET protein inhibitors to limit FcγR-mediated MNP activation.

We show that iBET dampened the expression of genes associated with IgG IC stimulation of MNPs and reduced the expression FcγRs on macrophages, decreasing their susceptibility to IgG-mediated activation. Interestingly, iBET increased macrophage phagocytosis, including phagocytic pathways independent of FcγR. iBET treatment also changed MNP morphology and resulted in a less adherent phenotype, prompting an assessment of its impact on DC migration. *In vitro*, IgG IC augmentation of DC chemotaxis to CCL19 was abrogated by addition of iBET. In keeping with this, systemic iBET treatment reduced IC-induced dermal DC mobilization *in vivo* and decreased their migration to draining lymph nodes, a DC-intrinsic effect. Together, our data confirm that iBET has a substantial impact on FcγR-mediated MNP activation and migration, and highlight therapeutic potential of bromodomain protein inhibitors to reduce tissue inflammation in antibody-mediated diseases, such as SLE.

## Materials and Methods

### Mice

Wild-type C57BL/6 mice, transgenic mice expressing enhanced yellow fluorescent protein (EYFP) under the control of the CD11c promoter, enhanced green fluorescent protein (EGFP) under the control of the human ubiquitin C promoter, and enhanced cyan fluorescent protein (ECFP) under the control of chicken β-actin promoter were obtained from Jackson Laboratories (Margate, UK). Fcγr2b-deficient mice on a C57BL/7 background (4) were provided by Jeff Ravetch (Rockefeller University, New York) and Silvia Bolland (National Institutes of Health, NIAID, Bethesda, MD). For all experiments, both male and female mice were used. For *in vivo* experiments, mice between the ages of 6 and 12 weeks were used. Mice were maintained in specific pathogen-free conditions at a Home Office-approved facility, with all procedures conducted in accordance with the United Kingdom Animals (Scientific Procedures) Act of 1986 and the GSK Policy on the Care, Welfare, and Treatment of Animals.

### iBET

I-BET151 (iBET) was supplied by GSK. For *in vitro* experiments, a stock solution of 10mM was prepared in DMSO solvent (Sigma) and was diluted and added to cell culture at the specified concentrations. For RNA sequencing experiments, BMDMs and BMDCs were treated with iBET at 5.0 and 3.3 μM respectively, added 30 minutes prior to immune complex stimulation. For *in vivo* experiments, iBET was administered at a concentration of 30 mg/kg, prepared by dissolving in normal saline containing 5% (v/v) DMSO and 10% (w/v) Kleptose HPB. For quantification of FcγR expression *in vivo*, mice were treated with intraperitoneal injection of iBET every 24 hours over 72 hours, followed by flow cytometry of harvested spleens.

### Immune Complexes

Endotoxin-free ovalbumin (1 mg/mL, Hyglos) was opsonized with a polyclonal rabbit anti-ovalbumin antibody (3.7 mg/mL, Sigma-Aldrich) at 37°C for 1 hour, at a 1:10 (v/v) ratio, to form insoluble immune complexes (IC), for stimulation of cells in culture or administration *in vivo*. Cells were harvested at defined time points and washed to remove free ICs. For RNA sequencing experiments, BMDMs and BMDCs were stimulated with immune complexes of ovalbumin for 4 hours. For phagocytosis assays, Alexa Fluor (AF)647-conjugated ovalbumin was used (Thermo Fisher), and conjugated with antibody as described above. For *in vivo* phagocytosis, 0.33 g/kg AF647-conjugated ovalbumin was opsonized with 3.2 g/kg polyclonal rabbit anti-ovalbumin antibody before injection. Phagocytic degradation products were identified by substituting Ova-647 with DQ^™^ Ovalbumin (Invitrogen), a conjugate that exhibits fluorescence only after proteolytic degradation.

### Culture of Mononuclear Phagocytes

For murine bone marrow-derived macrophages (BMDMs), bone marrow from femurs and tibias of mice were obtained by flushing with ice-cold sterile PBS and washed in ice-cold PBS. Bone marrow cells were then incubated in complete RPMI (cRPMI; 10% Hyclone FBS, 1% penicillin/streptomycin in RPMI-1640) supplemented with 100 ng/mL murine macrophage colony-stimulating factor (M-CSF, 100 ng/mL, PeproTech). Cells were cultured at 37°C over 5 days, with culture media being replaced on day 3. Adherent cells were harvested for downstream experiments and replated in cRPMI with M-CSF.

For murine bone marrow-derived dendritic cells (BMDCs), bone marrow cells were cultured in cRPMI supplemented with granulocyte-macrophage colony stimulating factor (GM-CSF, 40 ng/mL, PeproTech) over 8 days, with culture media being replaced on day 3 and 6. For BMDMs and BMDCs, cells were incubated with endotoxin-free ovalbumin (Hyglos) or immune-complexed ovalbumin for 24h at 37°C, on day 6 and 9 respectively, together with iBET at the specified concentration or DMSO control.

For murine peritoneal macrophages, the peritoneal cavity was flushed with 5 mL ice-cold sterile PBS. Cells harvested from peritoneal lavage were plated in cRPMI for 1 hour and adherent cells were used for downstream experiments.

Human monocyte-derived macrophages (moMac) were generated from the peripheral blood of healthy volunteers, obtained with informed consents under an IRB/EC approved protocol. Peripheral blood mononuclear cells (PBMCs) were isolated from Histopaque 1077 (Sigma-Aldrich) density separation, and monocytes enriched by negative selection using a MACS-based monocyte purification kit (Miltenyi Biotec). Purified PBMCs were cultured for 5-10 days in cRPMI with human M-CSF (0.1 μg/mL, PeproTech), supplemented every 72 hours, and subsequently stimulated with IC for downstream experiments.

### RNA Extraction and RNAseq Sample Preparation

BMDMs or BMDCs in culture were lysed using RLT plus buffer (QIAGEN), vortexed, snap frozen on dry ice, and stored at -80°C. To extract RNA from cell lysates, RNeasy plus micro kit (QIAGEN) were used according to the manufacturer’s instructions. Genomic DNA contamination was removed using Optimal DNA depletion columns (QIAGEN). Purified RNA was eluted in nuclease free water (Ambion) and stored at -80°C. To assess the quality and concentration of purified RNA, RNA pico chip (Applied Biosystems) on Bioanalyzer 2000 (Applied Biosystems) was used according to the manufacturer’s instructions. For all RNAseq experiments, samples had an RNA integrity number of >8. For library preparation, SMARTer^®^ stranded total RNAseq mammalian pico input kit (Takara) was used according to the manufacturer’s instructions. 5ng of total RNA was used for production of libraries. Library size was assessed using a High Sensitivity DNA chip (Applied Biosystems) on Bioanalyzer 2000 (Applied Biosystems) according to the manufacturer’s instructions. Concentration of the library was determined by qPCR using ROX low KAPA library quantification kit (Roche). Libraries were pooled at an equimolar concentration with up to 10 libraries per pool.

### RNA-Sequencing

For investigation of the effect of iBET treatment and immune complex stimulation on the transcriptome of murine BMDMs and BMDCs, we performed bulk RNA-sequencing (RNA-seq) using Hiseq 4000 (Illumina) on a 2 x 150bp sequencing run. Sequencing was performed by Genewiz. Pooled libraries were de-multiplexed by Genewiz using Casava (Illumina) before transfer of the data to the University of Cambridge. The Fastq files from libraries prepared using the Takara library prep were trimmed of the first 3 nucleotides of the R1 strand. Contaminating adaptor sequences and poor-quality bases removed (bases with a Phred 33 score of < 30) using Trim Galore! (Babraham bioinformatics). The Illumina library preps were only trimmed for quality. Sequencing quality of the resulting files was assessed using FastQC (Babraham bioinformatics). Fastq files were aligned to the mm10 genome using HISAT2.

Subsequent RNA-seq analysis was performed in the R statistical environment. Resulting data is available on GEO under accension numbers GSE200033 and GSE200226. Reads were counted and assigned to genes using the Featurecount function from the Rsubread package and differential expression analysis was performed using *DESeq2* with an appropriate design matrix according to the default workflow, and batch effects removed using the *sva* package. Figures were plotted using the *ggplot2*, *pheatmap*, and *EnhancedVolcano* packages. Gene Set Enrichment Analysis (GSEA, https://www.gsea-msigdb.org/gsea) was conducted using GSEA 4.1.0 according to developers’ instruction, using the pre-ranked option and classic setting. The rank metric for pre-ranked GSEA was calculated according to the following formula:


Rank metric=1/Pvalue∗(|LFC|/LFC)

Hallmark and Kegg gene sets were downloaded from Molecular Signature Database (MSigDB). Migratory DC gene sets were obtained from differentially expressed genes from previously published single cell RNA-sequencing analysis (GEO: GSE131957 and GSE137710) (36, 37).

### Quantitative Polymerase Chain Reaction

RNA extraction was performed using Ambion RNA PureLink kit (Life Technologies) according to the manufacturer’s instructions, from lysates of BMDMs. RNA concentration and purity were determined by Nanodrop spectrophotometry (Thermo Fisher) and subjected to cDNA synthesis using a High Capacity RNA-to-cDNA kit (Life Technologies). Quantitative polymerase chain reaction (qPCR) samples were performed in triplicate using Taqman reagents and the following TaqMan Gene Expression Assay primers and probes (Thermo Fisher). Primers used: *Gapdh* (Mm99999915_g1), *Hprt* (Mm03024075_m1), *Fcgr1* (Mm00438874_m1), *Fcgr2b* (Mm00438875_m1), *Fcgr3* (Mm00438882_m1), *Fcgr4* (Mm00519988_g1), *Il1b* (Mm00434228_m1), *Il6* (Mm00446190_m1), *Tnf* (Mm00443258_m1). qPCR was performed using the Viia 7 PCR machine (Life Technologies) and gene expression normalised to *Gadph* or *Hprt* using the 2^-ΔCt^ method.

### Flow Cytometry

Cells in culture, peritoneal lavage, splenocytes or lymph node cell suspensions were blocked with 0.5% heat-inactivated mouse serum for 15 minutes, followed by extracellular staining for 1 hour at 4°C using a combination of fluorophore-conjugated antibodies. Staining for CCR7 was performed at room temperature. Viability staining was performed using LIVE/DEAD™ Fixable Aqua Dead Cell Stain Kit (Life Technologies) or Zombie™ UV/Aqua Fixable Viability Dye (Biolegend) according to manufacturer’s instructions. Fells were fixed in PBS containing 1% formaldehyde, 0.02% sodium azide, 2% glucose. Cell counting was performed using 123count eBeads (eBioscience). Flow cytometry data collection was performed on a LSRFortessaTM (Becton Dickinson) flow cytometer and data analysed using FlowJo software (BD, version 10.6). Antibodies used are listed in **Supplementary Information**.

### Confocal Microscopy

Murine BMDMs and BMDCs were seeded onto glass cover slips sterilized with 70% ethanol prior to placement in 24 well culture plates, prior to addition of IC or iBET where appropriate. After incubation, cells were washed to remove free IC and fixed to cover slips by incubation with FACS FICS for 15 minutes. Cells were blocked and permeabilized in blocking buffer (0.1M TRIS, 1% BSA, 0.1% Triton X-100, 1% normal goat serum), and subsequently incubated with primary antibodies diluted in blocking buffer for 2 hours. Following washing with 0.1M TRIS, cells were incubated for 2 hours in appropriate secondary antibodies. Where relevant, slides were incubated with phalloidin diluted in blocking buffer for 1 hour. Cells were washed and mounted onto slides using VectaShield Hardset Mounting Medium with DAPI (Vector Labs). Images were acquired using a Zeiss 710 or Zeiss 780 confocal microscope and analysed using Imaris software (Oxford instruments). Antibodies used are listed in **Supplementary Information**.

### Phagocytosis of Apoptotic Cells

Thymi of C57BL/6 were harvested and passed through a 70 μm cell trainer. Thymocytes were labelled with cell tracker orange (ThermoFisher) according to manufacturer’s instructions, then rendered apoptotic *via* treatment with 1 μM dexamethasone (Sigma) for 17 hours (38). Murine BMDMs were pre-treated for 17 hours with iBET (0.5 μM) or DMSO control. Phagocytosis was assessed by flow cytometry, 4 hours after incubation of BMDMs with apoptotic thymocytes.

### *In Vivo* Immune Complex Peritonitis Model for Phagocytosis

Wild type C57BL/6 mice were injected with iBET (30 mg/kg.) or solvent control intraperitoneally, prepared as described above, with a second dose given 24 hours later. 1 hour later, Alexa Fluor 647-conjugated immune complexes or ovalbumin control were injected intraperitoneally. After 6 hours, mice were sacrificed, and peritoneal cavity resident immune cells were obtained by flushing with 3 mL of ice-cold sterile PBS (with 3% FBS). Peritoneal lavage was stained for subsequent flow cytometry analysis.

### *In Vivo* Kidney MNP Stimulation Assay for Phagocytosis

Wild-type C57/BL6 mice were injected intraperitoneally with iBET (30mg/kg) or solvent control, with a second dose given 24 hours later, followed by an intravenous dose of Alexa Fluor 647-conjugated immune complexes or ovalbumin control, injected *via* tail vein at a dose of 500 ng/g. After 60 minutes, mice were sacrificed and organs flushed with ice-cold PBS. For tissue dissociation of murine kidneys, organs were finely minced, and digested in RPMI-1640 medium containing 10mM Hepes, 1 mg/mL collagenase A (Roche), 0.1 mg/mL DNaseI (Roche) and 2% heat inactivated FCS (Sigma Aldrich) for 20 minutes. Tissue pieces were mechanically dissociated through a 70μm cell strainer and washed with PBS containing 2% FCS, and red blood cell lysis was performed using distilled H_2_O containing 0.83% (w/v) NH_4_Cl, 0.1% (w/v) NaHCO_3_, 100 mM EDTA. Single cell suspensions were subjected to a 44% v/v Percoll gradient (Sigma Aldrich) and washed thoroughly in ice-cold PBS for downstream analysis. For RNA extraction for qPCR, murine kidneys were submerged after harvest in RNAlater™ Stablisation Solution (Invitrogen) for storage, and later homogenised in lysis buffer using Precellys^®^ homogenisation technology and RNA extracted using PureLink RNA Mini Kit (Invitrogen).

### *In Vitro* 3D Chemotaxis Assay

Migration of BMDCs to a chemokine gradient was assessed as previously described by Haessler et al. (39). Briefly, BMDCs derived from transgenic mice expressing a fluorescent tag were stimulated with IC for 24 hours in the presence of iBET or DMSO control. iBET-treated cells were ECFP labelled and DMSO-treated cells EGFP labelled. After washing, cells were resuspended to a concentration of 10 × 10^6^ cells/mL and incorporated within 300 μL of a 1.5 mg/mL bovine collagen gel (20 μL 10x Minimum Essential Media Eagle (Sigma), 20 μL water, 10 μL 7.5% sodium bicarbonate solution (Sigma), 50 μL RPMI 1640, 150 μL 3mg/mL bovine collagen I (Sigma), 50 μL cell suspension in RPMI) loaded into a μ-Slide Chemotaxis chamber (Ibidi), according to the manufacturer’s instructions. The chamber was incubated for 15 minutes at 37°C, inverting once to ensure uniform cell distribution during collagen gelatination. Following incubation and solidification of the gel matrix, CCL19 (100 ng/mL, Peprotech) was applied to the chemoattractant solution reservoir and the sink reservoir was maintained base media. Migratory behaviour was imaged using time lapse confocal microscopy on an inverted Zeiss LSM 780 inverted confocal microscope with a 20X 0.9 NA objective, with stacked images taken every 45 to 60 seconds, over 2 to 4 hours.

### *In Vivo* Migration Assay: Intravital Imagine of Dermal DCs by Two Photon Microscopy

CD11cEYFP mice were anaesthetised with isoflurane and footpads were imaged at 915nm using a Zeiss 510 microscope, as previously described previously (40, 41). Briefly, a 70 μm-thick section of the dermis containing lymphatic vessels were scanned at 3 μm Z-steps every 45 seconds for 60 minutes, to generate time-lapse sequences. Imaging was alternated between left and right footpads over an 8 hour period to compare footpads in which 50 μL of ovalbumin or immune complexes had been injected 18 hours prior. Ovalbumin (0.1 mg/mL) was administered subcutaneously into the right footpad and immune complexes (50 μg Ova; 150 μg rabbit anti-Ova) to the left, so that animals acted as their own controls for immune complex stimulation. iBET or DMSO control was administered intraperitoneally two hours prior to ovalbumin or immune complex injection. Qdot^®^ A655 (Invitrogen Molecular Probes) was administered intravenously prior to imaging, to delineate dermal vasculature, where successful. The investigator performing the imaging was not blinded to information regarding treatment or stimulation.

### *In Vivo* Migration Assay: FITC Paint Model

Dermal DC migration was assessed using a FITC sensitization model, as previously described (20, 42). Ovalbumin or immune-complex ovalbumin was administered subcutaneously to the base of the tail, flank, or groin on the right and left side respectively of wildtype C57BL/6 mice, allowing for comparison of two experimental conditions within the same animal. At the same time, FITC (8 mg/mL, Sigma) was dissolved in equal volumes of acetone and dibutyl phthalate (Sigma) and 25 μL was applied to shaved skin over the site of IC administration. iBET (30 mg/kg) or solvent control was administered intraperitoneally thrice over a 48 hour period beginning at the time of IC administration. 48 hours after FITC application, draining inguinal lymph nodes and non-draining brachial lymph nodes were harvested for comparison. Lymph nodes were homogenized, passed through a 70 μm cell strainer, and digested in collagenase A (1 mg/mL, Roche) DNaseI (1 mg/mL, Roche), and 2% FCS in PBS for 20 minutes. The number of FITC+ cells were quantified by flow cytometry in harvested lymph nodes.

### Image Analysis for Chemotaxis and Migration Assays

Both *in vitro* and *in vivo* migration sequences were processed using Imaris software (Oxford Instruments, version 7.4). The Snapshot tool was used to generate time-lapse movies, still images and track histories. To characterize migration behaviour of individual DCs and obtain track statistics, all DC migration data was analysed using the surface tracking feature in Imaris. Specifically, x- and y-coordinates for each cell tracked were recorded to plot spatial trajectories, to obtain values for displacement and distance covered. Speed values were recorded over the entire sequence. The standalone version of Ibidi’s Chemotaxis and Migration Tool (Ibidi) was used to visualize tracks for *in vitro* movies. Track displacement was calculated in the x- and y-axes for the *in vitro* chemotaxis assay. Track length refers to the accumulated distance travelled by each cell over the observed period. The centre of mass represents the averaged point of all cell end points respective to the x- and y-axes.

### BMDC Transfer Model

Fluorescent labelled BMDCs were generated from transgenic EGFP or ECFP mice, as described above, and were stimulated with IC over 16 hours and treated with iBET or DMSO respectively. Cells were washed to remove free IC and injected subcutaneously into the flank or footpad of wild-type recipient C57BL/6 mice. 48 hours following BMDC transfer, draining and non-draining lymph nodes were harvested and fluorescently labelled DCs were quantified by flow cytometry.

### Statistical Analysis

With the expression of RNA-seq analyses, described above, statistical analysis was performed using Graphpad PRISM software (version 9.0). Data is expressed as mean ± SEM, mean with individual data points, or median with individual data points as indicated in figure legends. For comparisons, Wilcoxon matched-pairs signed rank test, nonparametric Mann-Whitney-U test or two-tail Student t-test were applied, as described in figure legends. * P < 0.05, ** P < 0.01, *** P < 0.001, **** P < 0.0001, NS = not significant. Illustration in **Figure 6** was made in Affinity Designer (version 1.9).

## Results

### iBET Variably Inhibits FcγR Cross-Linking-Associated Transcriptional Changes in MNPs

**Figure 1:**
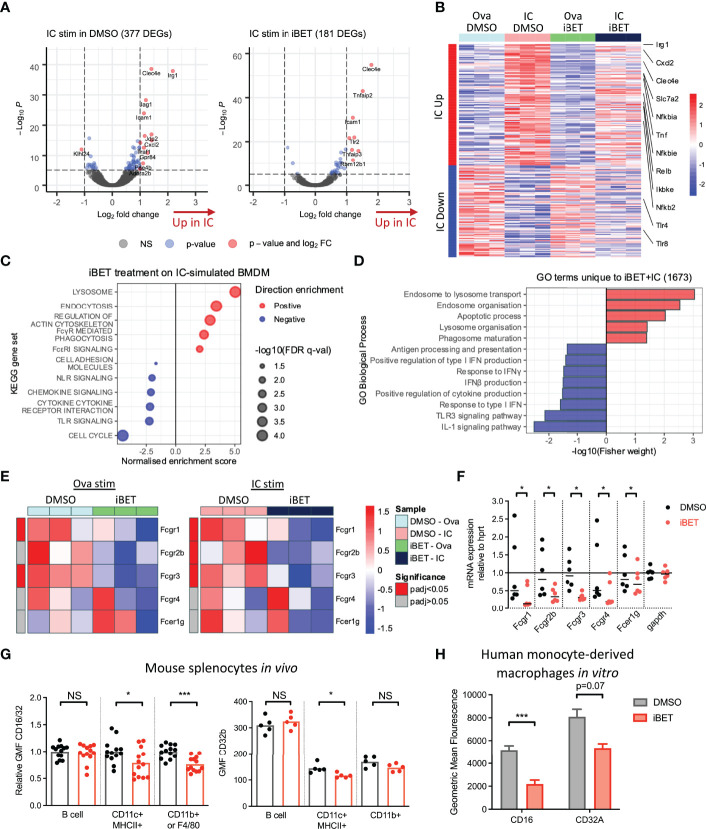
Gene expression changes induced by FcγR crosslinking are variably affected by iBET. **(A)** Volcano plots demonstrating DEGs stimulated by Ova-IC *vs* ovalbumin control, in the absence and presence of iBET, in murine BMDMs. (Adjusted P-value cut-off = 10e-6.) **(B)** Heat map of top 200 IC-induced DEGs with iBET treatment, with genes of interest involved in inflammatory signalling highlighted. **(C)** Gene set enrichment analysis (GSEA) of selected KEGG pathways affected by iBET treatment in IC-stimulated BMDMs; all FDR q-value < 0.05. **(D)** GO biological processes of DEGs unique to combination of IC stimulation and iBET treatment. **(E)** Heat map of *Fcgr* expression in BMDMs following iBET treatment, with Ova or Ova-IC stimulation. **(F)** qPCR of *Fcgr* expression in BMDMs following iBET treatment. Medians are shown, where points represent expression levels of BMDMs from individual mice (n=6). **(G)** Flow cytometry quantification of FcγR expression on splenic B cells, dendritic cells (DC) and macrophages *in vivo*, following systemic treatment with iBET for 3 days. Medians are shown for data representative of 4 independent experiments, points represent individual mice. **(H)** Flow cytometry quantification of FcγR expression on human monocyte-derived macrophages (moMac) *in vitro*, following treatment with iBET. Means ± SEM shown for data representative of 2 independent experiments from independent donors. BMDM RNA-seq data is representative of 3 biological replicates from independent mice. Significance testing using Wald test as described in DESeq2 **(E)**, Wilcoxon matched-pairs signed rank test **(F)**, Mann-Whitney U test **(G)**, and two-tailed Student’s t-test **(H)**.

To gain a global overview of the effect of iBET on FcγR-mediated MNP activation, we performed bulk RNA-sequencing (RNA-seq) on murine bone marrow-derived macrophages (BMDMs) following stimulation with insoluble IgG opsonized ovalbumin immune complexes (ICs) or ovalbumin control alone (Ova) for 4 hours (20), with iBET treatment or DMSO solvent control added 30 minutes prior to IC or Ova stimulation. 377 genes were significantly differentially expressed in IC+DMSO macrophages compared to 181 differentially expressed genes (DEGs) in IC+iBET cells (**Figure 1A**). Notably, the expression of several antimicrobial genes strongly driven by FcγR cross-linking, including *Irg1* and *Jag1* (43, 44), were abrogated by iBET treatment. Consistent with this, we observed a global dampening of top IC-inducible gene changes in iBET, including transcripts of known importance in inflammatory and NFκB signalling, such as *Tnf*, *Nfkbia*, *Nfkbie*, *Cxcl2*, *Ikbke*, etc. (**Figure 1B**). As anticipated, due to the extensive role of bromodomain proteins in transcriptional regulation (24, 25), treatment with iBET alone drove major changes in gene expression. 2263 genes were differentially expressed after just 4.5 hours of exposure to iBET, accounting for 9.3% of gene transcripts captured by RNA-seq (**Supplementary Figure 1A**).

To understand the biological significance of iBET-induced gene changes, we used gene set enrichment analysis (GSEA) (**Figure 1C** and **Supplementary Figure 1B**). iBET treatment down-regulated inflammatory signalling pathways, including interferon responses, ‘cytokine signalling’ and ‘toll-like receptor (TLR) signalling’ and ‘chemokine signalling’ in both IC and Ova-treated macrophages, with additional effects on genes relating to MNP phagocytosis (‘FcyR-mediated phagocytosis’) and morphology (‘regulation of actin cytoskeleton’). Gene Ontology (GO) biological processes enriched in DEGs unique to IC + iBET treatment (**Supplementary Figure 1C**) confirmed the anti-inflammatory effects of iBET, with negative enrichment of ‘response to IFNγ’ and ‘IL1 signalling pathway’ genes, but interestingly, induction of genes involved in phago-endocytic processes (**Figure 1D**).

Given effects on genes associated with FcyR-mediated phagocytosis, we next sought to profile whether iBET influenced the expression of FcγR genes themselves. RNA-seq of murine BMDMs showed reduced expression of activating receptors *Fcgr1* and *Fcgr3*, as well as the inhibitory *Fcgr2b* in iBET treated macrophages (**Figure 1E**), effects validated by qPCR (**Figure 1F**). Similarly, in bone marrow-derived dendritic cells (DC), iBET treatment also reduced *Fcgr* transcripts (**Supplementary Figure 2A**). We confirmed reduced cell-surface FcγR protein expression, (using a FcγRIIb/III-binding antibody, 2.4G2), on BMDM (**Supplementary Figure 2B**) and murine peritoneal macrophages (**Supplementary Figure 2C**) following *in vitro* iBET treatment. To explore the effects of iBET on FcγR expression *in vivo*, we treated mice systemically with iBET for 3 days. This resulted in a significant reduction in FcγRIIb/III expression on splenic CD11b+ macrophages and CD11c+ dendritic cells (DCs), with minimal effects on FcγRIIb expression, suggesting that the main effect of iBET *in vivo* is to reduce activating FcγR expression and to shift the activating-to-inhibitory (A/I) FcγR ratio to promote a less inflammatory state (**Figure 1G** and **Supplementary Figure 2D**). To demonstrate relevance in humans, we generated macrophages from peripheral blood monocytes, and similarly found a reduction in activating FcγR expression, particularly FcγRIII, following iBET treatment (**Figure 1H** and **Supplementary Figure 2E**).

### iBET Increases Macrophage Phagocytosis

**Figure 2:**
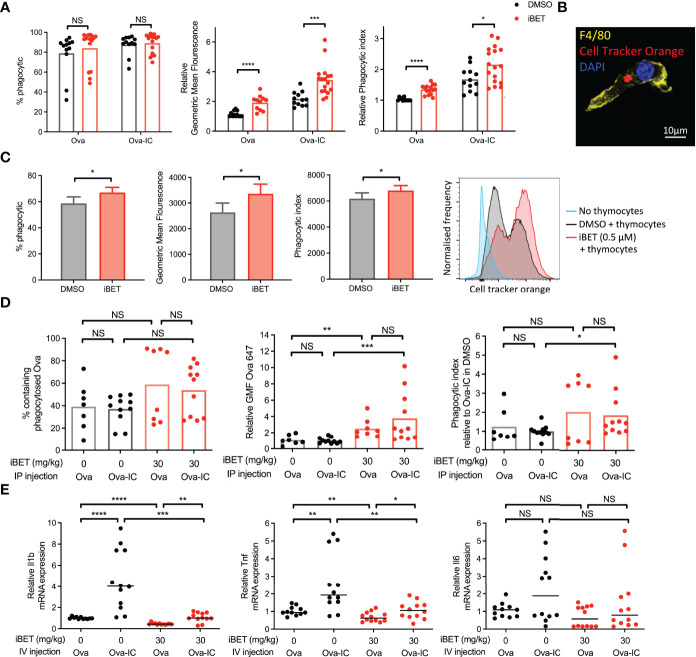
iBET alters macrophage phagocytosis. **(A)** Flow cytometry quantification for phagocytosis of fluorescent-labelled Ova-AF647 by murine BMDMs after 16 hours. Phagocytic index is the geometric mean fluorescence (GMF) of Ova+ gate (phagocytic macrophages). Means ± SEM shown for data representative of 5 independent experiments, normalized to DMSO-treated BMDMs in Ova, to allow comparison across experiments. **(B)** Representative image of murine BMDM with phagocytosed fluorescent-labelled (cell tracker orange) apoptotic thymocytes after 4 hours. **(C)** Flow cytometry quantification for phagocytosis of apoptotic thymocytes by BMDMs. Means ± SEM shown for data representative of 2 independent experiments. **(D)** Flow cytometry quantification for peritoneal macrophage accumulation of fluorescent-labelled immune complexed or soluble antigens *in vivo*. Medians are shown for data representative of 3 independent experiments, points represent individual mice. Relative values to untreated Ova-IC stimulated mice reported due to interexperimental variation in Ova-AF647 fluorescence. **(E)** qPCR of inflammatory cytokine expression in whole kidney tissue following iBET treatment and IC stimulation. Medians are shown from 2 independent experiments, where points represent expression levels from individual mice. Significance testing using two-tailed Student’s t-test **(A, C)**, Mann-Whitney U test **(D, E)**.

Our analysis of the effect of iBET on the transcriptome of BMDMs (**Figures 1C, D** and **Supplementary Figure 1B**) suggested that phagocytic processes may be enhanced in MNPs, despite the reduced expression of FcγRs we identified (**Figures 1E-H**). To test this functionally, we quantified *in vitro* phagocytosis of fluorescent-labelled Ovalbumin (Ova) or IgG opsoni**s**ed Ova by BMDMs, and observed that iBET treatment significantly increased the per cell uptake of both Ova and Ova-IC, as evidenced by an increase in MFI (**Figure 2A**). Macrophage phagocytosis is not only important for clearing circulating immune complexes, but is also required to remove apoptotic cells. Indeed, reduced apoptotic cell clearance is thought to promote the breakdown of tolerance to nuclear antigens presented on the surface of apoptotic blebs, contributing to lupus pathogenesis (45, 46). We therefore generated apoptotic cell tracker-labelled thymocytes (38) and added them to BMDMs that were pre-treated with iBET or DMSO control. iBET treatment resulted in an increased accumulation of apoptotic cell debris within macrophages compared with DMSO-treated cells (**Figures 2B, C**).

Next, we sought to determine the effect of iBET on macrophage phagocytosis *in vivo*. Mice were pre-treated with iBET or solvent control, followed by the administration of Ova or Ova-IC intraperitoneally (**Supplementary Figure 3A**). 6 hours post-IC challenge there was a similar proportion of Ova-positive macrophages in iBET-treated mice compared with controls however, the per cell uptake of both Ova and Ova-IC was significantly higher in the iBET group (**Figure 2D**). iBET treatment did not affect the frequency of peritoneal cells (**Supplementary Figure 3B**), nor phagocytosis by peritoneal DCs (**Supplementary Figure 3C**).

Increased levels of Ova fluorescence may result from increased antigen uptake or delayed lysosomal degradation leading to accumulation of fluorescent material. To probe this point, we generated insoluble IgG immune complexes with fluorescent DQ-Ova, a compound that fluoresces only upon proteolytic degradation in the lysosome. This did not indicate any delayed appearance of DQ-Ova in the presence of iBET (**Supplementary Figure 3D**). Indeed, at 15 minutes, increased accumulation was already evident. Interestingly, at later timepoints, the difference in DQ signal between iBET and DMSO-treated macrophages did not increase, as one might expect if there was impaired lysosomal degradation. Altogether, this data supports an increase in Fc-mediated phagocytosis in the presence of iBET.

We sought to extend our observations to a tissue context relevant to SLE, specifically the effect of circulating IgG immune complex on kidney resident macrophages. Kidney macrophages may arise from yolk-sac progenitors or be monocyte-derived, characterised by F4/80^high^ CD11b^int^ and F4/80^int^ CD11b^high^ expression respectively (47), and differ in their functional characteristics (48). Mice were pre-treated with iBET or solvent control, followed by intravenous administration of Ova or Ova-IC (**Supplementary Figure 4A**), where both populations of kidney macrophages have been previously shown to uptake immune complex (49). The administration of intravenous IC led to an increased frequency of F4/80^int^ CD11b^high^ kidney monocyte-derived macrophages (**Supplementary Figure 4B**), robust phagocytosis of Ova-IC compared with Ova alone, with little effect of iBET treatment (**Supplementary Figures 4C, D**). Despite this avid phagocytosis of circulating IgG IC, administration of iBET attenuated the IC-associated increase in pro-inflammatory cytokine gene expression in the kidney, including *Il1b* and *Tnf* transcripts (**Figure 2E**). Altogether, these data suggest that iBET may enhance the removal of both IgG ICs and apoptotic cells, without a corresponding increase in pro-inflammatory signalling, both potentially beneficial to attenuate lupus pathogenesis.

### iBET Alters MNP Morphology and Adhesion

**Figure 3:**
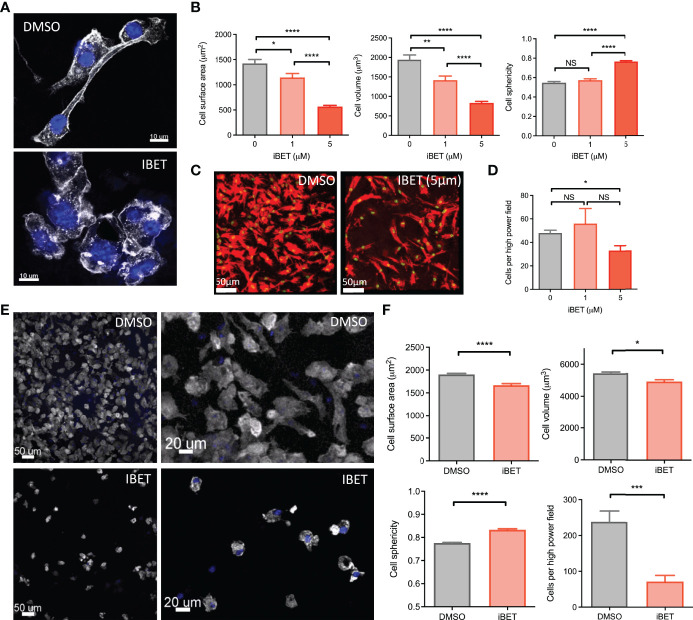
iBET alters MNP morphology and adhesion. **(A)** Representative images showing effect of iBET treatment on murine BMDM morphology *in vitro*. DAPI stain shown in blue, phalloidin in white. **(B)** Quantification of BMDM morphology by confocal microscopy of murine BMDMs following treatment with iBET for 24 hours. **(C)** Representative image change in BMDM adhesion after iBET treatment *in vitro*; phalloidin (red), DAPI (green). **(D)** Cells per high powered field observed after iBET treatment. Means ± SEM shown for data representative of 5 high-powered fields per condition. Means ± SEM shown for data representative of 5 high-powered fields per condition **(A–D)**. **(E)** Representative images showing effect of iBET treatment on murine BMDC morphology and adhesion *in vitro*. DAPI stain shown in blue, phalloidin in white. **(F)** Quantification of BMDC adhesion and morphology by confocal microscopy of murine BMDCs following treatment with iBET (3.3μM) for 24 hours. Means ± SEM shown for data representative of 10 high-powered fields per condition. Significance testing using two-tailed Student’s t-test **(B, D, F)**.

Additional gene pathways regulated by iBET in BMDM included ‘*regulation of actin cytoskeleton’* (**Figure 1C** and **Supplementary Figure 1B**). Consistent with, we observed morphological changes in iBET-treated BMDMs *in vitro*, with a reduction in dendritic processes (**Figure 3A**), a decrease in cell surface area and volume, and an increase in sphericity (**Figure 3B**). At higher concentrations, iBET treatment resulted in a decrease in adherent cells observed per high power field (**Figures 3C, D**). Similar effects on cell morphology were observed in murine bone marrow-derived dendritic cells (BMDCs) following iBET treatment, with decreased cell adhesion and increased sphericity (**Figures 3E, F**), but no significant effects on cell viability (**Supplementary Figure 4E**). Overall, these iBET-induced morphological changes might reasonably be expected to influence cell motility and chemotaxis, processes of particular importance for tissue DCs, enabling migration from peripheral tissues to draining lymph nodes, critical for antigen presentation to, and spatial co-localisation with, CD4 T cells (9, 10).

### iBET Limits Chemokine-Directed and IC-Induced DC Chemotaxis

**Figure 4:**
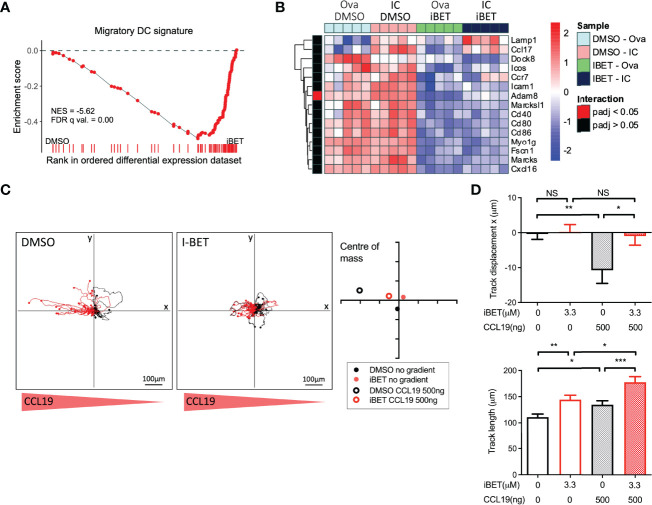
iBET limits DC migration *in vitro*. **(A)** GSEA of gene signature specific to migratory DCs, curated from scRNAseq data, in iBET treated murine BMDCs. Gene signature derived from Brown et al. (2019), GEO: GSE137710. **(B)** Heat map of expression of selected genes involved in DC migration and maturation. **(C)** Representative migration tracks of Ova-IC stimulated BMDCs over 2 hours, and average centre of mass in a 3D collagen matrix with or without a 500ng CCL-19 gradient. Red track marks represent cells with final displacement in the direction of the chemokine gradient. **(D)** Selected chemotaxis parameters from **(C)**. Means ± SEM shown for data representative of 3 independent experiments, including at least 180 tracked BMDCs for each condition. RNA-seq data is representative of 5 biological replicates from independent mice. Significance testing using Student’s t-test.

GSEA of RNAseq of BMDCs treated with iBET (**Supplementary Figures 5A, B**) demonstrated negative enrichment of ‘chemokine signalling’ and ‘cell adhesion molecules’ gene sets with iBET treatment, in both Ova and Ova-IC treated DCs (**Supplementary Figure 5C**). Furthermore, *in vivo* migratory DC gene signatures curated from publicly available single-cell RNA-seq (scRNAseq) datasets of tissue cells (36, 37) were also negatively enriched in iBET-treated BMDCs compared with DMSO-treated cells (**Figure 4A** and **Supplementary Figure 5D**). Indeed, several genes known to be important for DC migration and maturation (36), including *Dock8* (a Cdc42-specific guanine nucleotide exchange factor that is critical for interstitial DC migration) (50), several chemokine receptors, and co-stimulatory molecules (51), were down-regulated in iBET-treated DCs (**Figure 4B** and **Supplementary Figure 5E**).

A major mechanism by which tissue DCs migrate to lymph nodes is *via* CCR7-dependent chemotaxis to CCL19 and CCL21 (18, 19), a process stimulated by FcyR cross-linking on DCs (20). iBET treatment of IC-stimulated DCs embedded within a three-dimensional collagen matrix (52) led to reduced chemotaxis towards a CCL-19 gradient compared with DMSO-treatment, although iBET had no effect on overall DC movement or migration speed (**Figures 4C, D** and **Supplementary Figure 5F**). Consistent with our observations on the effect of iBET on MNP chemotaxis, we found that iBET impaired recruitment of monocyte-derived macrophages to the kidney in our model of systemic IC administration (**Supplementary Figure 4B**).

**Figure 5:**
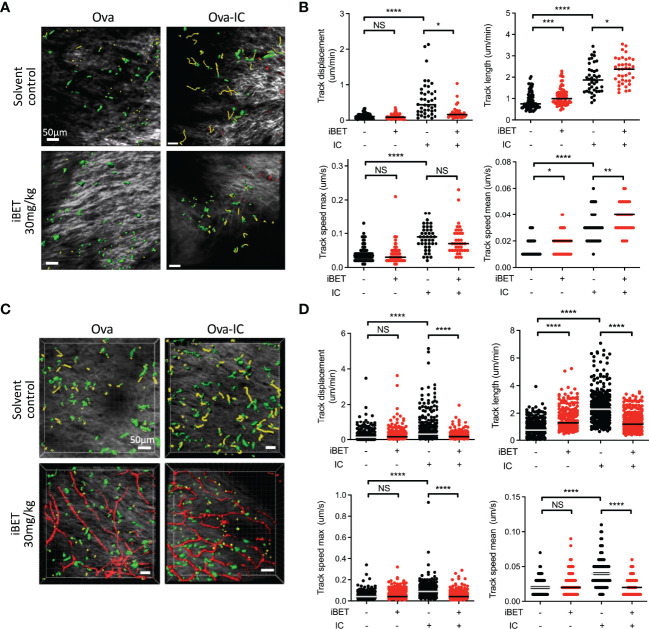
iBET impairs IC-associated DC chemotaxis *in vivo.* **(A)** Representative images showing movement of CD11c-YFP labelled dermal DCs in mice footpads by two-photon microscopy. Following treatment with iBET or solvent control, CD11c-YFP reporter mice were injected with Ova in one hind footpad and Ova-IC in the contralateral footpad, with imaging under isoflurane anaesthesia 18 hours later. Green shows DCs, yellow shows representative migration tracks of respective DCs. **(B)** Quantification of chemotaxis of dermal DCs in mice footpads. **(C)** Representative images showing movement of CD11c-YFP labelled dermal DCs and **(D)** quantification of chemotaxis in footpads of FcγRIIb -/- mice. Blood vessels labelled with Qdot^®^ probe (red), shown where successful. For all, medians are shown for data representative of 2 independent experiments, points show individual tracked dermal DCs. Significance testing using Mann-Whitney U test.

To determine if iBET inhibited IC-stimulated chemotaxis of endogenous DCs *in vivo* we used intravital two-photon microscopy to assess the movement of dermal DCs in WT and *Fcgr2b* ^-/-^ CD11cEYFP mice (20), the latter a model for enhanced activating FcyR signalling (3). Systemic iBET treatment significantly decreased IC-induced DC track displacement, reflecting directional mobilization, in both strains, with variable effects on DC movement and speed, consistent with an inhibition of chemotaxis but not chemokinesis (**Figures 5A-D**).

### iBET Impairs IC-Stimulated DC Migration Towards Lymph Nodes

**Figure 6:**
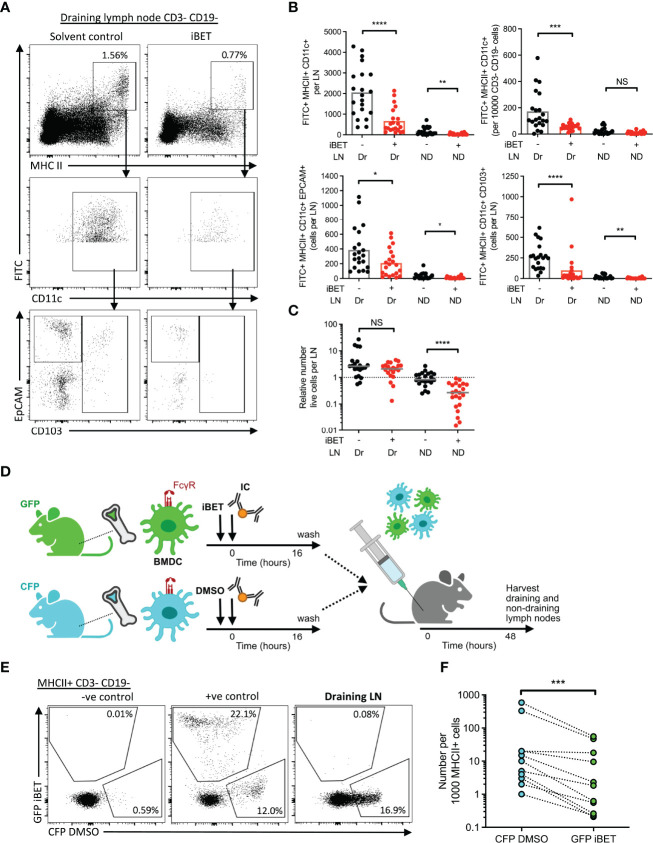
iBET impairs IC-stimulated DC migration to lymph nodes. **(A)** Representative flow cytometry plots of draining lymph nodes of mice from FITC paint model. FITC paint was applied topically to shaved skin of mice to label dermal DCs, stimulated and treated with IC and iBET with appropriate controls, and draining lymph nodes were harvested 48 hours later. **(B)** Flow cytometry quantification of dermal DCs from draining and non-draining lymph nodes from FITC paint model. Medians are shown for data representative of 4 independent experiments, points show individual mice. **(C)** Lymph node cell number in FITC paint model following iBET treatment. **(D)** Diagram of experimental set up for murine BMDC transfer model. BMDC were cultured from fluorescent-labelled mice and treated with iBET or DMSO followed by transfer to wild-type mice. Recipient mice were culled 48 hours alter and lymph nodes were harvested. **(E)** Representative flow cytometry plot of CFP and GFP staining of DCs in draining lymph nodes. **(F)** Flow cytometry quantification of DC composition in draining lymph nodes. Data shown is representative of 10 mice from 3 independent experiments. Significance testing using Mann-Whitney U test **(B, C)** and Wilcoxon matched-pairs sign rank test **(E)**.

Next, we asked whether iBET could prevent dermal DCs from completing their migration to draining lymph nodes, using an established FITC paint model (20) to label dermal DCs, followed by local administration of IC and simultaneous systemic treatment the mice with iBET (**Supplementary Figure 6A**). At 48 hours post-IC stimulation, there was a significant reduction in the frequency of FITC^+^ MHC class II^high^ CD11c^+^ DCs in draining lymph nodes in iBET-treated mice compared to controls. This reduction was observed in both tissue-resident EPCAM+ Langerhans cell, as well as CD103+ conventional DCs (**Figures 6A, B** and **Supplementary Figure 6B**). We also noted a reduction in FITC-labelled DCs in non-draining lymph nodes, and that mice treated systemically with iBET had smaller lymph nodes. This was associated with a reduction in live cells in non-draining lymph nodes (**Figure 6C**), suggesting iBET may affect homeostatic leucocyte migration to lymph nodes, even in the absence of an inflammatory stimulus.

The reduction in DC migration observed in iBET-treated animals could be due to direct cell-intrinsic effects on DCs, or due to effects on cells involved in governing chemotaxis, such as chemokine-producing stromal cells. We therefore utilized a DC transfer model in which BMDCs from green fluorescent protein (GFP)-expressing mice and cyan fluorescent protein (CFP)-expressing mice were stimulated *in vitro* with IC, and simultaneously treated with iBET or DMSO respectively (**Figure 6D** and **Supplementary Figure 7A**). An equal mixture of iBET and control DCs (**Supplementary Figure 7B**) were administered subcutaneously to wild-type mice, and the number of DCs reaching draining lymph nodes quantified. We observed a significant reduction in the proportion of iBET-treated DCs reaching the draining lymph node compared with DMSO-treated DCs (**Figures 6E, F**).

## Discussion

BET family proteins play an important role in controlling the transcription of proinflammatory and immunoregulatory genes (31), evidenced by the action of the BET protein inhibitor JQ1 in suppressing immune signalling gene networks with nodes converging on RELA-, JUN- and STAT1-mediated transcriptional responses (53). Here, we interrogated the effects of the bromodomain inhibitor iBET (GSK525762A) on macrophages and DCs and their responses to IgG IC, demonstrating attenuation of FcyR crosslinking-associated inflammatory gene expression (including those involved in NFκB signalling), and chemotaxis, but an enhancement of antigen internalisation. Bromodomain inhibitors have been shown to have marked anti-inflammatory effects in many models of autoimmune and inflammatory disease (32–35, 53, 54). Consistent with our findings, the BET protein BRD4 has been shown to be required for the complete trans-activation of RELA in proinflammatory NFκB signalling in other contexts (55). In inflammatory renal disease, inhibition of bromodomain proteins may reduce NFκB-mediated tissue damage (56). Further, BET protein inhibitors irreversibly suppressed the development of type I diabetes in NOD mice by promoting an anti-inflammatory phenotype in pancreatic macrophages, rather than infiltrating T cells, *via* modulation of the NFκB genes (54).

BET protein inhibition has also been showed to modulate JAK and STAT kinase-dependent inflammatory signalling. In human monocyte-derived macrophages, transcription of STAT targets downstream of cytokine stimulation, such as TNFα, interferon (IFN)-β and IFN-γ, were suppressed by BET inhibition, without affecting activation of the STAT protein itself (57). In a mouse model of non-alcoholic steatohepatitis, inhibition of BET proteins significantly reduces the expression of STAT1-dependent IFN-γ in liver tissue (58). Our transcriptomic data show that several cytokine pathways that rely on downstream JAK-STAT signalling were suppressed by treatment with iBET, including type I and II interferon, interleukin (IL)-2 and IL-6. Additionally, BET protein inhibition reduced IC-induced production of inflammatory cytokines in tissue-resident macrophages, which has also been previously shown in the context of LPS stimulation (35, 59), thus subduing an auto-inflammatory loop potentiated by autocrine cytokine signalling.

Importantly, we found that iBET reduces the expression of FcγRs that engage IgG IC *in vitro* and *in vivo*, with a greater effect on activating FcγRs versus the inhibitory FcγRIIb. The resulting shift in the A/I FcγR ratio on the surface of MNPs towards a more inhibitory state would be predicted to increase the activation threshold of MNPs encountering IgG-IC, potentially contributing to the attenuated change in gene expression observed in response to FcγR cross-linking. This suggests that iBET could limit inflammation by increasing macrophage activation thresholds, and in DCs, the FcγR A/I ratio has also been shown to control the magnitude of T cell activation (60).

Beyond effects on FcγR-induced inflammatory gene expression, our transcriptomic analysis also indicated that iBET may alter several additional MNP functions, including antigen internalisation and migration. Strikingly, we observed increased per cell uptake of soluble protein antigen, large, insoluble immune complex, and apoptotic cells in iBET-treated macrophages, the latter an established source of nuclear antigens contributing to pathogenic ICs in SLE (61). The effect of iBET in dampening activating FcyR and pro-inflammatory gene expression, combined with this increase in phagocytosis may well promote non-inflammatory clearance of ICs by tissue-resident MNPs. In DCs, where endo-phagocytic processes precede antigen processing and presentation, iBET may facilitate the clearance of IgG-opsonised antigens while preventing downstream activation and maturation, including co-stimulatory molecule expression (62, 63), which we observed were decreased in iBET-treated BMDC. Of note, our study did not probe the precise mode of antigen internalisation affected by iBET, which may be *via* endocytosis, fluid-phase pinocytosis or phagocytosis (64–69). This may be of interest for future study.

Our observations that iBET altered the ‘dendritic’ morphology of cells in culture mirrors observations in neurons reported previously (70). However, we additionally demonstrate that iBET inhibited DC chemotaxis and migration from skin to draining lymph nodes following IgG IC stimulation, important processes that enable autoantigens to be presented to CD4 T cells to propagate pathological responses. Of note, BRD4 has been described to be important in the movement of other cell types, including malignant cell metastases (71, 72). Our observations on the effects of iBET on MNP trafficking may also contribute to previously described effects in attenuating leucocyte accumulation in LPS-induced vascular and lung inflammation (73). Of note, abnormalities in monocytes and DCs have been observed in patients with lupus (74–77) and we have previously shown that engagement of activating FcγRs on tissue DCs by autoantibody-containing IC in lupus leads to the migration of DCs to draining lymph nodes (20). Furthermore, the lupus-associated polymorphism in human FCGR2B (rs1050501) (78) that results in receptor dysfunction (7, 8) is associated with increased CCR7 expression on DCs following IgG IC stimulation, driving enhanced migration to the T cell zone of lymph nodes, propagating autoimmunity and inflammation. Hence, iBET inhibition of IgG IC-driven DC migration may represent a useful therapy to break this pro-inflammatory feed-forward loop in antibody-mediated autoimmune diseases such as lupus. Future studies might also address the effects of iBET on monocyte migration and recruitment.

Despite the role of BET proteins in epigenetic regulation, epigenetic mechanisms underlying BET inhibitors and control of inflammation remain poorly characterised. In the context of TLR4 ligation, suppression of NFκB directed super-enhancer dependent pro-inflammatory gene transcription is thought to underlie anti-inflammatory effects observed. FcγR crosslinking is also known to induce epigenetic changes, where chromatin remodelling has been described to underlie IC-induced IL-10 secretion in macrophages (79) and contribute to dysregulated inflammatory responses in rheumatoid arthritis (80). However, more detailed investigation of the precise epigenetic changes associated with FcγR-driven inflammation is required to optimize the application of bromodomain inhibitors.

In conclusion, our study provides mechanistic insight into the potential therapeutic benefit of iBET in the setting of antibody-driven inflammation. We show that bromodomain inhibitors modulate IC driven MNP responses altering surface A/I FcγR ratios and reducing pro-inflammatory gene networks whilst increasing the phagocytosis of IC and apoptotic cells. Importantly, iBET inhibited DC chemotaxis and limited migration to lymph nodes, with potential pro-tolerogenic effects in reducing antigen presentation to CD4 T cells. Overall, our work emphasises the potential of modulating epigenetic processes for the treatment of IC-mediated autoimmune diseases.

## Data Availability Statement

The RNA sequencing data reported in this paper have been deposited in the Gene Expression Omnibus (GEO) database under accension codes GSE200033 and GSE200226.

## Ethics Statement

The studies involving human participants were reviewed and approved by IRB/EC. The patients/participants provided their written informed consent to participate in this study. The animal study was reviewed and approved by REC12/EE/0446.

## Author Contributions

GB designed and performed experiments, data analysis and interpretation. CL performed data analysis and interpretation, analysed gene expression data, and wrote the manuscript. JF provided guidance on experiment design and data analysis. RM and CJ performed experiments. NS and RP was involved in conception and design of the study. MC supervised the project, designed and performed experiments, and wrote the manuscript. All authors contributed to the article and approved the submitted version.

## Funding

This study received funding from GSK for RNA sequencing experiments. GSK was not involved in the study design, collection, analysis, interpretation of data, the writing of this article or the decision to submit it for publication. GB was funded by a Wellcome Translational Medicine and Therapeutics PhD grant (102728/z/13/z). CL was funded by the Gates Cambridge scholarship and University of Cambridge School of Clinical Medicine. JF, RM, CJ, and MC were supported by the National Institute of Health Research (NIHR) Cambridge Biomedical Research Centre and the NIHR Blood and Transplant Research Unit in Organ Donation (NIHR BTRU-2014-10027), and MC by a Medical Research Council New Investigator Research Grant (MR/N024907/1) and an Arthritis Research UK Cure Challenge Research Grant (21777).

## Conflict of Interest

Author NS and RP are employees of and shareholders in GlaxoSmithKline (GSK). This study received funding from GSK. GSK had the following involvement with the study: funded RNA sequencing.

The remaining authors declare that the research was conducted in the absence of any commercial or financial relationships that could be construed as a potential conflict of interest.

## Publisher’s Note

All claims expressed in this article are solely those of the authors and do not necessarily represent those of their affiliated organizations, or those of the publisher, the editors and the reviewers. Any product that may be evaluated in this article, or claim that may be made by its manufacturer, is not guaranteed or endorsed by the publisher.

## Supplementary Material

Supplementary Figure 1 Supporting BMDM RNA-seq data. **(A)** Volcano plot demonstrating DEGs with iBET treatment at baseline conditions (Ovalbumin stimulation). **(B)** Gene set enrichment analysis (GSEA) of selected Hallmarks and KEGG pathways affected by IC stimulation and iBET treatment. Colour represents normalized enrichment score (NES), size represents FDR-q value; all FDR q-value < 0.05. **(C)** Venn diagram showing common DEGs affected by IC stimulation and iBET treatment. *Effect of iBET* refers to iBET *vs* DMSO treatment in ovalbumin or IC-stimulated BMDMs. *Effect of IC* refers to IC *vs* ovalbumin stimulation in iBET or DMSO control-treated BMDMs.

Supplementary Figure 2 Supporting FcγR expression data. **(A)** Heatmap for Fcgr expression in BMDCs following iBET treatment, with Ova or Ova-IC stimulation. **(B)** Flow cytometry quantification and representative flow plot of FcγR expression on murine BMDMs treated with iBET. Means ± SEM shown for data representative of 2 independent experiments. **(C)** Flow cytometry quantification and representative flow plot of FcγR expression on murine peritoneal macrophages *in vitro*, following treatment with iBET. Means ± SEM shown for data representative of 3 independent experiments. **(D)** Representative flow plots from , FcγR expression on splenocytes *in vivo*. **(E)** Representative flow plots from , FcγR expression on human moMacs. Significance testing using one-way ANOVA test **(B)**, and two-tailed Student’s t-test **(C)**.

Supplementary Figure 3 Supporting data for intraperitoneal immune complex peritonitis model. **(A)** Flow cytometry gating for peritoneal lavage. Mice were given immune-complexed Ova or Ova control by intraperitoneal injection and sacrificed 6 hours later with peritoneal lavage for quantification of inflammatory cell recruitment and assessment of phagocytosis. **(B)** Flow cytometry quantification of peritoneal lavage cell composition. **(C)** Flow cytometry quantification of peritoneal dendritic cell phagocytosis following iBET treatment. **(D)** Time-course of DQ-Ova-IC degradation by murine BMDMs following iBET treatment. Data in **(A-C)** show medians representative of 3 independent experiments, points represent individual mice. Relative values to untreated Ova-IC stimulated mice reported due to interexperimental variation in Ova-AF647 fluorescence. Significance testing using Mann-Whitney U test.

Supplementary Figure 4 Supporting data for intravenous immune complex administration to assess effect of iBET on kidney MNPs. **(A)** Flow cytometry gating for kidney MNPs. Mice were given immune-complexed Ova or Ova control by intravenous injection and sacrificed 1 hour later with renal tissue processed for assessment. **(B)** Flow cytometry quantification of frequency of renal macrophage populations. **(C, D)** Flow cytometry quantification of frequency of phagocytic renal macrophages **(C)**, and phagocytic index **(D)**, following iBET treatment. All data show medians representative of 2 independent experiments, points represent individual mice. Frequency of renal macrophage populations are normalised to the mean of population counts in control kidneys (no IC/iBET). **(E)** Treatment with iBET does not affect cell viability. Means ± SEM shown for data representative of 3 independent experiments. Significance testing using Mann-Whitney U test **(B-D)** and one way ANOVA test **(E)**.

Supplementary Figure 5 Supporting BMDC migration data *in vitro*. **(A)** Volcano plots demonstrating DEGs stimulated by Ova-IC, in the absence and presence of iBET, in murine BMDCs. (Adjusted P-value cut-off = 10e-6.) Venn diagram shows overlapping DEGs induced by IC stimulation in presence or absence of iBET treatment. **(B)** Volcano plot demonstrating DEGs with iBET treatment at baseline conditions (Ovalbumin stimulation). **(C)** GSEA of selected KEGG pathways affected by iBET stimulation, without and without IC stimulation, in murine bone marrow derived dendritic cells (BMDC). All FDR q-value < 0.05. **(D)** GSEA for gene expression signatures representative of migratory DCs, from scRNAseq data, following iBET treatment, in murine BMDCs. Gene signatures derived from GEO: GSE131957 and GSE137710. **(E)** Heat map of murine chemokine receptor gene expression. **(F)** Additional parameters from chemotaxis parameters of Ova-IC stimulated BMDCs in collagen matrix with or without a 500ng CCL-19 gradient. Means ± SEM shown for data representative of 2 independent experiments, including at least 180 tracked BMDCs for each condition. Significance testing using Student’s t-test.

Supplementary Figure 6 Supporting data for BMDC migration by FITC painting. **(A)** Flow cytometry gating of draining lymph nodes from FITC paint model. **(B)** Data from , with y-axis presented in log-scale.

Supplementary Figure 7 Supporting data for BMDC migration by fluorescent BMDC transfer. **(A)** Flow cytometry gating of draining lymph nodes from fluorescent BMDC transfer model. **(B)** Flow cytometry of murine BMDCs pre-transfer, showing CFP and GFP fluorescence.
