# Supplementary material for: Bromodomain Inhibitors Modulate FcγR-Mediated Mononuclear Phagocyte Activation and Chemotaxis
Source: Front Immunol. 2022 May 10;13:885101. doi: 10.3389/fimmu.2022.885101 (PMC9127238; doi:10.3389/fimmu.2022.885101)
Supplement: Supplementary file 8 [file Table_1.docx]

Supplementary Material

# Supplementary Tables

**Supplementary table 1.1: Antibodies**

| Antigen | | Fluorophore | | Clone | | Species/Isotype | | Supplier | | Product number | | Dilution | |
| --- | --- | --- | --- | --- | --- | --- | --- | --- | --- | --- | --- | --- | --- |
| Mouse: | |  | |  | |  | |  | |  | |  | |
| CCR7 | | PE | | 4B12 | | Rat/IgG2a, κ | | Biolegend | | 120106 | | 1:100 | |
| CD103 | | PE | | M290 | | Rat/IgG2a, κ | | BD Biosciences | | 557495 | | 1:200 | |
| CD11b | | eFluor® 450 | | M1/70 | | Rat/IgG2b, κ | | Biolegend | | 101224 | | 1:200 | |
| CD11c | | eFluor® 450 | | N418 | | Armenian hamster/IgG | | eBioscience | | 48-0114-82 | | 1:200 | |
| CD11c | | PE | | N418 | | Armenian hamster/IgG | | eBioscience | | MA5-16878 | | 1:200 | |
| CD11c | | PECy7 | | N418 | | Armenian hamster/IgG | | Biolegend | | 117318 | | 1:200 | |
| CD16/32 | | PECy7 | | 2.4G2 | | Rat/IgG2b, κ | | BD Biosciences | | 560829 | | 1:200 | |
| CD16/32 | | PE | | 2.4G2 | | Rat/IgG2b, κ | | BD Biosciences | | 553145 | | 1:200 | |
| CD19 | | PE | | eBio1D3 (1D3) | | Rat/IgG2a, κ | | eBioscience | | 12-0193-83 | | 1:200 | |
| CD19 | | PECy7 | | eBio1D3 (1D3) | | Rat/IgG2a, κ | | eBioscience | | 25-0193-82 | | 1:200 | |
| CD19 | | PERCP Cy5.5 | | 6D5 | | Rat/IgG2a, κ | | Biolegend | | 115534 | | 1:200 | |
| CD19 | | BV785 | | 6D5 | | Rat/IgG2a, κ | | Biolegend | | 115543 | | 1:200 | |
| CD3 | | BV605 | | 17A2 | | Rat/IgG2b, κ | | Biolegend | | 100237 | | 1:200 | |
| CD3 | | BV785 | | 17A2 | | Rat/IgG2b, κ | | Biolegend | | 100231 | | 1:200 | |
| CD32b | | APC | | AT130-2 | | Mouse/IgG2a, κ | | eBioscience | | 17-0321-82 | | 1:200 | |
| CD45 | | FITC | | 30-F11 | | Rat/IgG2b, κ | | eBioscience | | 11-0451-85 | | 1:200 | |
| CD45 | | UV395 | | HI30 | | Mouse/IgG1, κ | | BD Biosciences | | 56427 | | 1:200 | |
| EpCAM | | APC | | G8.8 | | Rat/IgG2a, κ | | eBioscience | | 17-5791-82 | | 1:200 | |
| F4/80 | | FITC | | BM8 | | Rat/IgG2a, κ | | eBioscience | | 11-4801-85 | | 1:200 | |
| F4/80 | | BV605 | | BM8 | | Rat/IgG2a, κ | | Biolegend | | 123133 | | 1:200 | |
| Ly-6C | | PERCP Cy5.5 | | HK1.4 | | Rat/IgG2c, κ | | eBioscience | | 45-5932-80 | | 1:200 | |
| Ly-6G (Gr-1) | | PECy7 | | RB6-8C5 | | Rat/IgG2b, κ | | eBioscience | | 25-5931-82 | | 1:200 | |
| Ly-6G (Gr-1) | | APC-eFluor® 780 | | RB6-8C5 | | Rat/IgG2b, κ | | eBioscience | | 47-5931-82 | | 1:200 | |
| MHC Class II-1 | | eFluor® 450 | | AF6-120.1 | | Mouse/IgG2a, κ | | eBioscience | | 48-5320-82 | | 1:200 | |
| MHC Class II (I-A/I-E) | | BV650 | | M5/114.15.2 | | Rat/IgG2b, κ | | Biolegend | | 107641 | | 1:400 | |
| MHC Class II (I-A/I-E) | | A700 | | M5/114.15.2 | | Rat/IgG2b, κ | | eBioscience | | 56-5321-82 | | 1:200 | |
| Human: |  | |  | |  | |  | |  | |  | |  |
| CD16 | ef450 | | CB16 | | Mouse/IgG1, κ | | EBioscience | | 48-0168-41 | | 1:200 | |  |
| CD19 | APC Cy7 | | SJ25C1 | | Mouse/IgG1, κ | | BD Biosciences | | 557791 | | 1:25 | |  |
| CD3 | AF488 | | HIT3a | | Mouse/IgG2a, κ | | Biolegend | | 300320 | | 1:100 | |  |
| CD32a | unconjugated | | | | Polyclonal goat IgG | | R&D | | AF1330 | | 1:100 | |  |

**Supplementary table 1.2: Other stain reagents**

| Product | Fluorophore | Supplier | Product Number | Dilution |
| --- | --- | --- | --- | --- |
| Ovalbumin | Alexa Fluor™ 647 | Invitrogen | O34784 | As described |
| LIVE/DEAD™ Fixable Aqua Dead Cell Stain Kit |  | Invitrogen | L34957 | 1:300 |
| 123count eBeads™ Counting Beads |  | eBioscience | 01-1234-42 | 25000-50000 beads/test |
| Zombie UV™ Fixable Viability Dye |  | Biolegend | 423107 | 1:500 |
| Zombie Aqua™ Fixable Viability Dye |  | Biolegend | 423101 | 1:500 |
| VectaShield Hardset Mounting Medium with DAPI |  | Vector Labs | H-1500-10 |  |
| QDot 655 probe |  | Invitrogen Molecular Probes |  | 1:5 |
| Phalloidin | Alexa Fluor™ 568 | Invitrogen | A12380 | 1:100 |
| Phalloidin | Alexa Fluor™ 647 | Invitrogen | A22287 | 1:50 |
| Anti-Goat IgG | Alexa Fluor™ 488 | Thermo Scientific | A32814 | 1:100 |
| DQ™ Ovalbumin | BODIPY FL^®^ | Invitrogen | D12053 | As described |
